# Redetermination of the crystal structure of bis­(tri-2-pyridyl­amine)­iron(II) bis­(perchlorate), and a new refinement of the isotypic nickel(II) analogue: treatment of the perchlorate anion disorder

**DOI:** 10.1107/S2056989018005601

**Published:** 2018-04-17

**Authors:** Zouaoui Setifi, Fatima Setifi, Necmi Dege, Rafika El Ati, Christopher Glidewell

**Affiliations:** a Département de Technologie, Faculté de Technologie, Université 20 Août 1955-Skikda, BP 26, Route d’El-Hadaiek, Skikda 21000, Algeria; b Laboratoire de Chimie, Ingénierie Moléculaire et Nanostructures (LCIMN), Université Ferhat Abbas Sétif 1, Sétif 19000, Algeria; c Ondokuz Mayıs University, Arts and Sciences Faculty, Department of Physics, 55139 Atakum–Samsun, Turkey; d Laboratoire de Chimie Appliquée et Environnement, (LCAE), Faculté des Sciences, Université Mohamed Premier, BP 524, 60000, Oujda, Morocco; e School of Chemistry, University of St Andrews, St Andrews, Fife KY16 9ST, UK

**Keywords:** crystal structure, polypyridyl complexes, redetermination, perchlorate anion disorder, hydrogen bonding, supra­molecular assembly

## Abstract

The redetermination of the structure of the title compound, [Fe(C_15_H_12_N_4_)_2_](ClO_4_)_2_, (I), confirms the structure previously reported [Kucharski *et al.* (1978*a* ▸). *Aust. J. Chem*. **31**, 53–56], but models the perchlorate over four sets of atomic sites, rather than using just one set of sites as in the original report. The supra­molecular assembly, not reported previously, takes the form of a complex three-dimensional framework built from C—H⋯O hydrogen bonds. The isotypic nickel(II) analogue, [Ni(C_15_H_12_N_4_)_2_](ClO_4_)_2_, (III), has been refined using the original data set [Wang *et al.* (2011 ▸). *Acta* Cryst. E**67**, *m*78], again using a four-component disorder model for the anion, rather than a two-component model as in the original report, leading to more satisfactory Cl—O distances and O—Cl—O angles.

## Abstract 2

The crystal structure of bis­(tri-2-pyridyl­amine)­iron(II) bis­(perchlorate) has been redetermined, and that of the isotypic bis­(tri-2-pyridyl­amine)­nickel(II) bis­(perchlorate) complex has been rerefined. In each case, the perchlorate anion is disordered over four sets of atomic sites, and the ions are linked by C—H⋯O hydrogen bonds to form a supra­molecular three-dimensional framework.

## Chemical context   

The crystal structure of bis­(tri-2-pyridyl­amine)­iron(II) bis(perchlorate) was reported a number of years ago (Kucharski *et al.*, 1978*a* ▸), as was that of the isotypic Co^II^ analogue (Kucharski *et al.*, 1978*b* ▸). In each of these structures, the metal centre lies at a centre of inversion, with a single perchlorate anion occupying a general position: the metal–N distances are consistent with a low-spin configuration in the Fe^II^ complex, but a high-spin configuration in the Co^II^ complex (Kucharski *et al.*, 1978*a* ▸,*b* ▸). In each structure the unique perchlorate anion was modelled using a single set of atomic sites, but the anisotropic displacement parameters give a clear indication of unmodelled disorder in this species.

As a part of our continuing study of the structural and magnetic properties of iron complexes containing poly-pyridyl ligands (Setifi *et al.*, 2013*a* ▸,*b* ▸, 2014 ▸, 2016 ▸, 2017 ▸), we have now re-investigated the structure of compound (I), using a new data set. However, we have used the *P*2_1_/*n* setting of space group No. 14 rather than *P*2_1_/*a*, as used in the original report, as this setting has a smaller value of β, 98.716 (7)°, than the *P*2_1_/*a* setting where β is 121.38 (3)° (Kucharski *et al.*, 1978*a* ▸). The sample used here was prepared under solvothermal conditions in a 4:1 water/ethanol mixture, in the presence of potassium 1,1,3,3-tetra­cyano-2-eth­oxy­propenide.
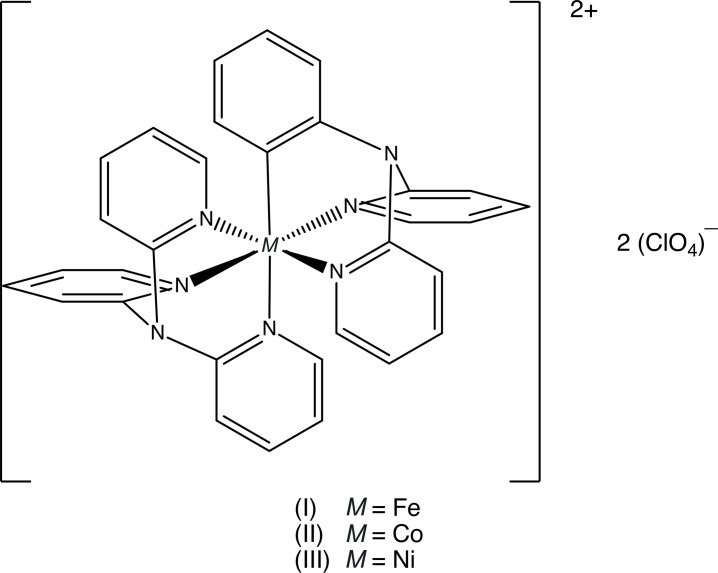


The Ni^II^ analogue (III) is isotypic with compounds (I) and (II), although in this case the refinement was conducted (Wang *et al.*, 2011 ▸) in space group *P*2_1_/*n* rather than in the alternative P2_1_/*a* setting used for (I) and (II) (Kucharski *et al.*, 1978*a* ▸,*b* ▸). In their refinement of the Ni complex, the perchlorate anion was modelled using two sets of atomic sites, having occupancies 0.528 (19) and 0.472 (19). However, the reported Cl—O distances range from 1.2136 (4) to 1.5356 (6) Å while the reported O—Cl—O angles lie in the range 96.48 (3)–118.284 (12)°; both of these ranges seem to be too wide to be correct, and accordingly we have undertaken a new refinement of this structure using the original data set (Wang *et al.*, 2011 ▸).

## Structural commentary, and treatment of the perchlorate anion disorder   

**Figure 1:**
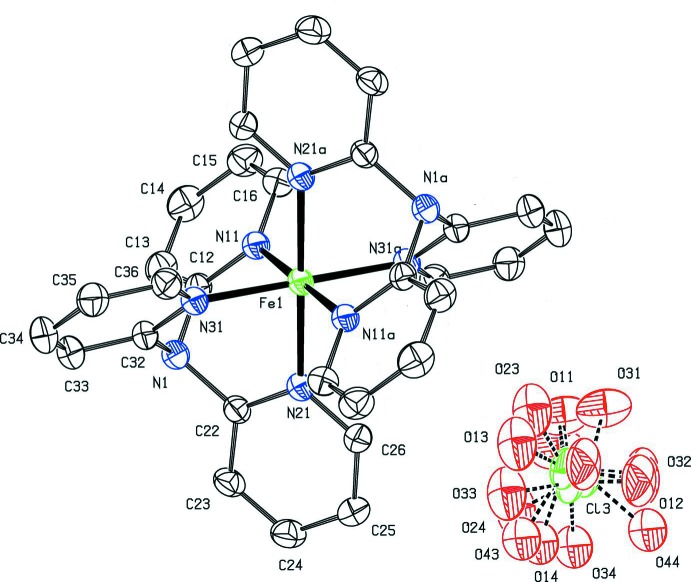
The ionic components of compound (I), with atom labelling and displacement ellipsoids drawn at the 30% probability level. For clarity, the H atoms and the symmetry-equivalent anion have been omitted, and unmarked atoms and atoms marked ‘a’ are at the symmetry position (−*x* + 1, −*y* + 1, −*z* + 1)*.*

As noted above, the metal atom in compound (I) lies on a centre of inversion, selected here as that at (0.5, 0.5, 0.5), and the organic ligand is tridentate with the ligating atoms N11, N21 and N31 (Fig. 1 ▸) adopting a facial configuration: the Fe—N distances are 1.983 (2), 1.970 (3) and 1.982 (3) Å, respectively, fully consistent with low-spin Fe^II^ (Orpen *et al.*, 1989 ▸). However, when the refinement used only a single set of atomic sites for the perchlorate anion, this resulted in very large, prolate displacement ellipsoids for the O atoms, indicative of positional disorder. Accordingly, this anion was modelled using, in succession, two, three or four sets of atomic sites and only for the last could the anisotropic displacement parameters be regarded as satisfactory: the final refined values of the occupancies are 0.415 (3), 0.267 (3), 0.256 (3) and 0.061 (3) (Fig. 1 ▸).

**Figure 2:**
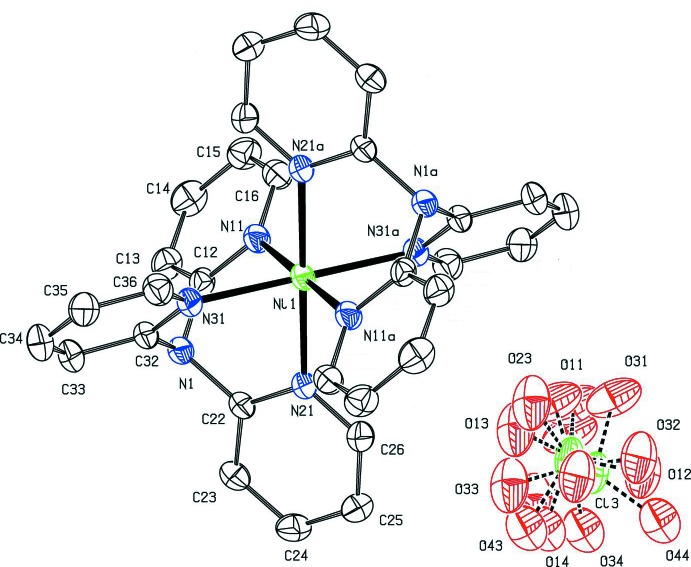
The ionic components of compound (III), with atom labelling and displacement ellipsoids drawn at the 30% probability level. For clarity, the H atoms and the symmetry-equivalent anion have been omitted, and unmarked atoms and atoms marked ‘a’ are at the symmetry position (−*x* + 1, −*y* + 1, −*z* + 1).

For the isotypic Ni^II^ complex (III) (Fig. 2 ▸), the same set of multi-component disorder models as employed for (I) were investigated, but only the four-component model gave satisfactory displacement parameters: the refined occupancies of the perchlorate components are 0.424 (3), 0.280 (3), 0.244 (3) and 0.052 (3), very similar to those for (I). The resulting range of Cl—O distances in (III) is 1.401 (5)–1.438 (5) Å and that of the O—Cl—O angles is 107.1 (4)–112.5 (5)°, both more satisfactory that those obtained in the original two-component model (Wang *et al.*, 2011 ▸).

## Supra­molecular features   

**Figure 3:**
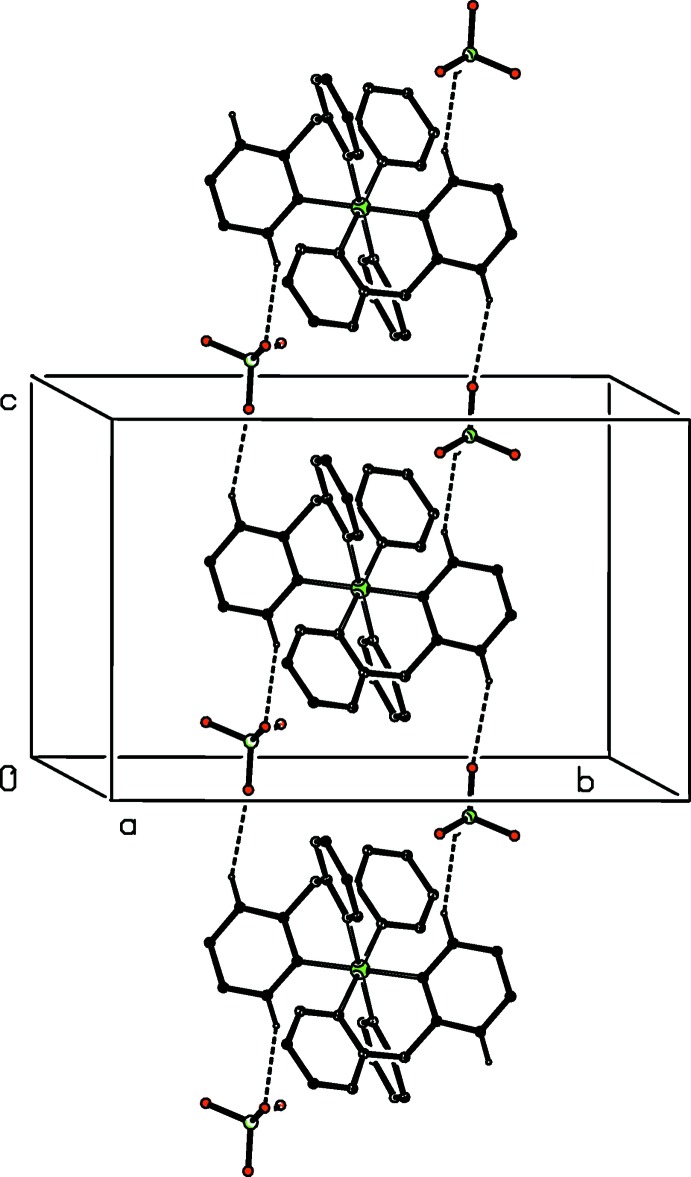
Part of the crystal structure of compound (I), showing the formation of a hydrogen-bonded ribbon running parallel to the [001] direction. For the sake of clarity, only the major disorder component of the anion is shown and the H atoms not involved in the motif shown have been omitted.

**Figure 4:**
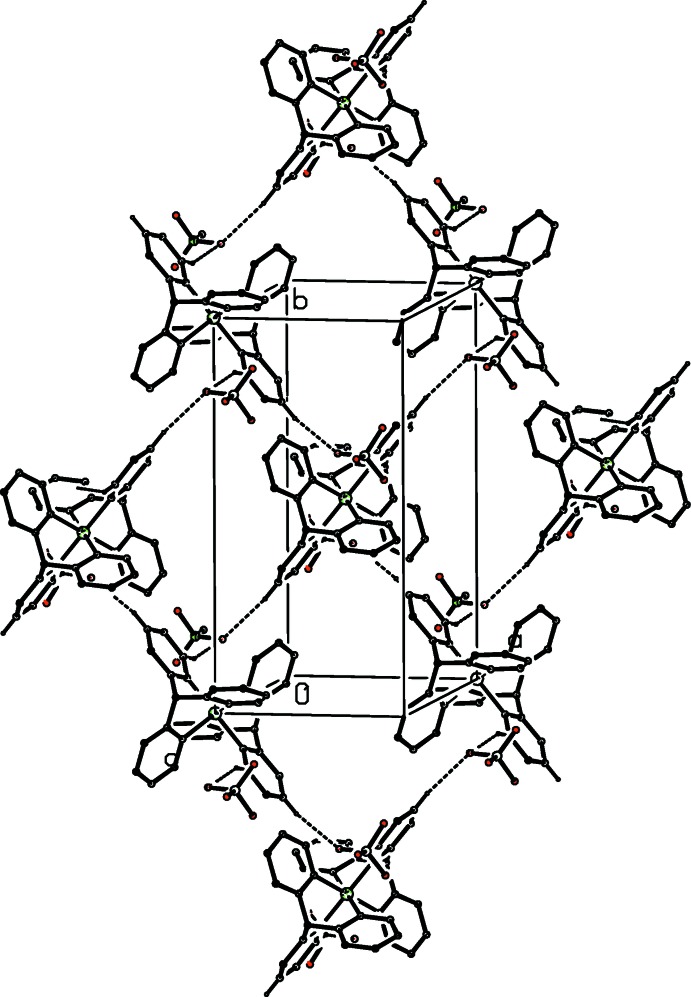
Part of the crystal structure of compound (I), showing the formation of a hydrogen-bonded sheet lying parallel to (101). For the sake of clarity, only the major disorder component of the anion is shown and the H atoms not involved in the motif shown have been omitted.

There are neither C—H⋯N nor C—H⋯π(pyrid­yl) hydrogen bonds in the crystal structure of compound (I); nor are there any π–π stacking inter­actions. The supra­molecular assembly is dependent on C—H⋯O hydrogen bonds (Table 1 ▸): although the anion disorder introduces complexity, the close similarity between the patterns of the inter­actions involving the different disorder components means that, only those of the dominant component, based on atom Cl1, need be considered, as entirely similar aggregation arises from the other components also. There are just three C—H⋯O hydrogen bonds involving the major component, one of which lies within the selected asymmetric unit: in combination, these three hydrogen bonds link the ions into a three-dimensional supra­molecular framework whose formation is readily analysed in terms of two sub-structures (Ferguson *et al.*, 1998*a* ▸,*b* ▸; Gregson *et al.*, 2000 ▸). In the simpler sub-structure, the two hydrogen bonds involving atoms C23 and C26 as the donors and atoms O12 and O13 as the acceptors link the ions into a ribbon running parallel to the [001] direction and in which 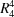
(22) rings centred at (0.5, 0.5, *n*) link the metal complexes centred at (0.5, 0.5, 0.5 + *n*), where *n* represents an integer in each case (Fig. 3 ▸). In the second substructure, the two hydrogen bonds having atom O13 as the acceptor, link the ions into a sheet lying parallel to (101); see Fig. 4 ▸. The combination of the [001] chain and the (101) sheet is sufficient to generate a three-dimensional supra­molecular framework. For compound (III), the pattern of the hydrogen bonds (Table 2 ▸) is very similar to that in (I), as is the supra­molecular assembly. It is inter­esting to note that no C—H⋯O hydrogen bonds were mentioned in the original report on (I) (Kucharski *et al.*, 1978*a* ▸), possibly because only a decade or so earlier, the very idea of such inter­actions had been authoritatively dismissed (Donohue, 1968 ▸): perhaps more surprising is the absence of any mention of these inter­actions in the original report on compound (III) (Wang *et al.*, 2011 ▸).

## Database survey   

As noted above, the cobalt analogue (II) of compounds (I) and (III) is isotypic with them (Kucharski *et al.*, 1978*b* ▸).
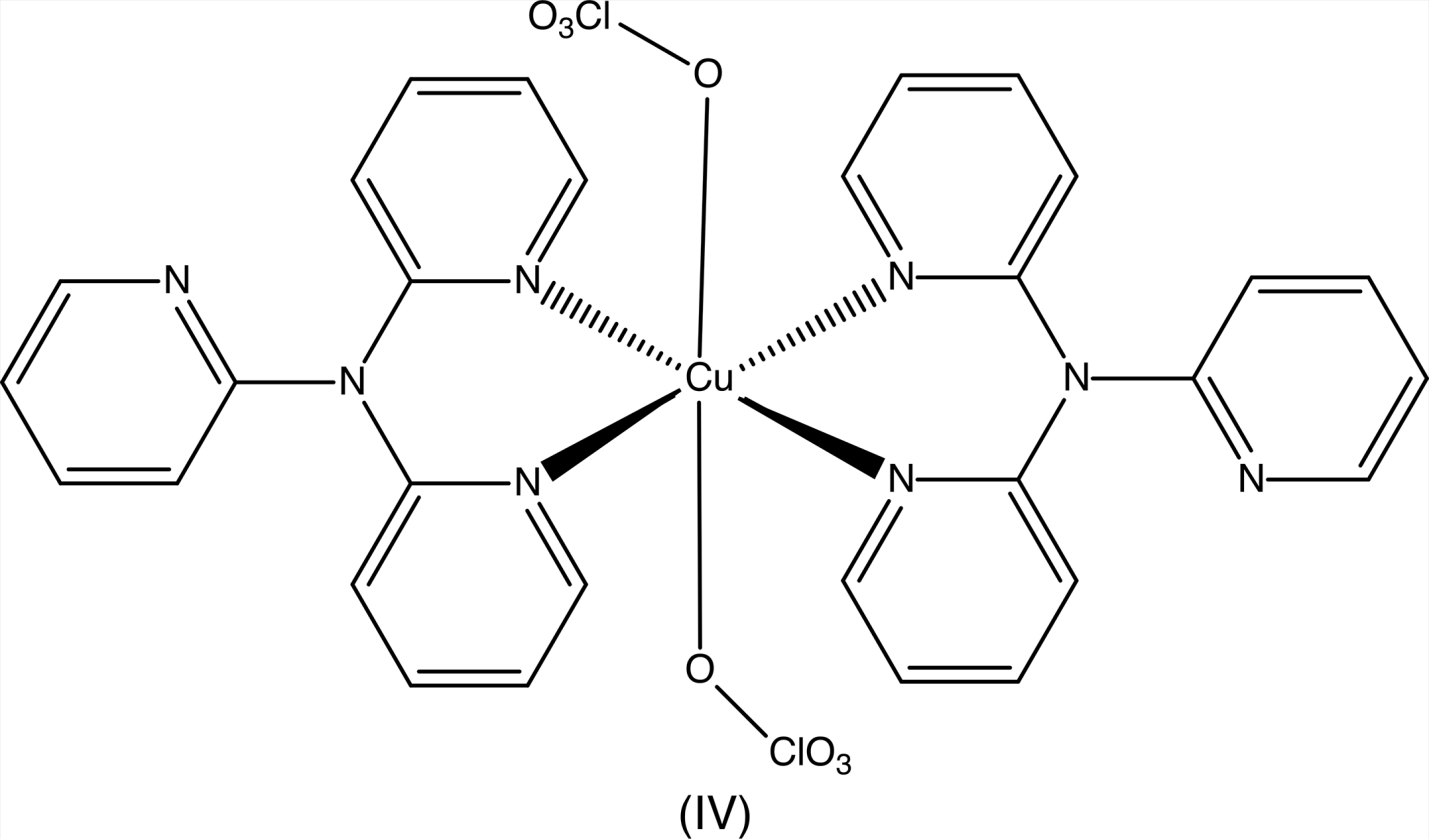


The corresponding copper complex (IV) has the same composition as compounds (I)–(III) and, like them, crystallizes in space group *P*2_1_/*n* with *Z*′ = 0.5 (Boys *et al.*, 1992 ▸) but its constitution is different: the organic ligand is only bidentate, giving a square planar CuN_4_ array with Cu—N distances of 1.992 (3) and 2.006 (3) Å; the usual (4 + 2) coordination of Cu^II^ is completed by two weakly-coordinated perchlorato ligands with a Cu—O distance of 2.593 (8) Å. By contrast, in the corresponding bis­(tri­fluoro­methane­sulfonate) salt the anion plays no role in the metal coordination, where the bidentate amine ligands form a distorted tetra­hedral geometry (Pérez *et al.*, 2009 ▸).

## Synthesis and crystallization   

For the synthesis of compound (I), a mixture of iron(II) sulfate hepta­hydrate (56 mg, 0.2 mmol), tri-2-pyridyl­amine (62 mg, 0.2 mmol) and potassium 1,1,3,3-tetra­cyano-2-eth­oxy­propenide (45 mg, 0.2 mmol) in water–ethanol (4:1 *v*/*v*, 20 ml) was placed in a Teflon-lined autoclave and heated at 423 K for 48 h. The autoclave was then allowed to cool to ambient temperature. Red prismatic crystals of the title compound were collected by filtration, washed with water and dried in air (yield 25%).

## Refinement   

Crystal data, data collection and structure refinement details are summarized in Table 3 ▸. All H atoms were located in difference-Fourier maps. They were then treated as riding atoms in geometrically idealized positions with C—H = 0.93 Å and *U*_iso_(H) = 1.2*U*_eq_(C). For the minor disorder components of the perchlorate anion in each compound the bonded distances and the 1,2 non-bonded distances were restrained to be the same as the corresponding distances in the dominant component, subject to s.u.s of 0.005 Å and 0.01°, respectively: in addition, the anisotropic displacement parameters for corresponding atom sites were constrained to be the same. Subject to these conditions, the refined values of the anion occupancies were 0.415 (3), 0.267 (3), 0.256 (3) and 0.061 (3) in (I) and 0.424 (3), 0.280 (3), 0.244 (3) and 0.052 (3) in (III). In the final analysis of variance for (I) there were two large values of *K* = [mean(*F*_o_^2^)/mean(*F*_c_^2^)], 11.399 for the group of 368 very weak reflections having *F*_c_/*F*_c_(max) in the range 0.000 < *F*_c_/*F*_c_(max) < 0.007, and 3.057 for the group of 312 very weak reflections having *F*_c_/*F*_c_(max) in the range 0.008 < *F*_c_/*F*_c_(max) < 0.0014; the corresponding value for (III) was 23.606 for 417 reflections having *F*_c_/*F*_c_(max) in the range 0.000 < *F*_c_/*F*_c_(max) < 0.007.

## Supplementary Material

Crystal structure: contains datablock(s) global, I, III. DOI: 10.1107/S2056989018005601/su5436sup1.cif

Structure factors: contains datablock(s) I. DOI: 10.1107/S2056989018005601/su5436Isup2.hkl

Structure factors: contains datablock(s) III. DOI: 10.1107/S2056989018005601/su5436IIIsup3.hkl

CCDC references: 1836078, 1556391

Additional supporting information:  crystallographic information; 3D view; checkCIF report

## Figures and Tables

**Table 1:** Hydrogen-bond geometry (Å, °) for (I)

*D*—H⋯*A*	*D*—H	H⋯*A*	*D*⋯*A*	*D*—H⋯*A*
C14—H14⋯O21^i^	0.93	2.38	3.11 (2)	136
C14—H14⋯O34^i^	0.93	2.54	3.470 (17)	173
C15—H15⋯O22^ii^	0.93	2.44	3.24 (3)	143
C15—H15⋯O32^ii^	0.93	2.31	3.14 (3)	147
C23—H23⋯O12^iii^	0.93	2.58	3.412 (10)	150
C23—H23⋯O22^iii^	0.93	2.52	3.357 (14)	150
C23—H23⋯O32^iii^	0.93	2.53	3.347 (12)	147
C24—H24⋯O13^iv^	0.93	2.60	3.496 (12)	163
C24—H24⋯O33^iv^	0.93	2.53	3.334 (17)	145
C24—H24⋯O42^iv^	0.93	2.21	3.10 (4)	161
C26—H26⋯O13	0.93	2.51	3.375 (12)	155
C26—H26⋯O33	0.93	2.56	3.289 (17)	135
C33—H33⋯O42^iii^	0.93	2.30	3.21 (4)	164

Symmetry codes: (i) 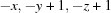; (ii) 
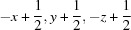
; (iii) 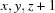; (iv) 
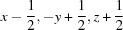
.

**Table 2:** Hydrogen-bond geometry (Å, °) for (III)

*D*—H⋯*A*	*D*—H	H⋯*A*	*D*⋯*A*	*D*—H⋯*A*
C14—H14⋯O21^i^	0.93	2.41	3.174 (17)	139
C14—H14⋯O34^i^	0.93	2.55	3.477 (14)	174
C14—H14⋯O43^i^	0.93	2.38	3.23 (4)	151
C15—H15⋯O12^ii^	0.93	2.52	3.289 (18)	140
C15—H15⋯O22^ii^	0.93	2.37	3.14 (3)	140
C15—H15⋯O32^ii^	0.93	2.57	3.37 (3)	144
C23—H23⋯O12^iii^	0.93	2.58	3.428 (8)	152
C23—H23⋯O22^iii^	0.93	2.53	3.376 (13)	152
C24—H24⋯O33^iv^	0.93	2.49	3.301 (13)	146
C24—H24⋯O42^iv^	0.93	2.12	2.97 (3)	152
C26—H26⋯O13	0.93	2.48	3.380 (11)	162
C26—H26⋯O33	0.93	2.57	3.331 (13)	140

Symmetry codes: (i) 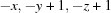; (ii) 
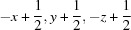
; (iii) 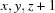; (iv) 
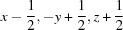
.

**Table 3:** Experimental details

	(I)	(III)
Crystal data		
Chemical formula	[Fe(C_15_H_12_N_4_)_2_](ClO_4_)_2_	[Ni(C_15_H_12_N_4_)_2_](ClO_4_)_2_
*M*_r_	751.32	754.18
Crystal system, space group	Monoclinic, *P*2_1_/*n*	Monoclinic, *P*2_1_/*n*
Temperature (K)	296	296
*a*, *b*, *c* (Å)	8.3251 (7), 17.4731 (11), 11.0495 (9)	8.360 (4), 17.570 (8), 11.165 (5)
β (°)	98.716 (7)	99.542 (5)
*V* (Å^3^)	1588.8 (2)	1617.3 (13)
*Z*	2	2
Radiation type	Mo *K*α	Mo *K*α
μ (mm^−1^)	0.71	0.83
Crystal size (mm)	0.42 × 0.21 × 0.12	0.22 × 0.15 × 0.10

Data collection		
Diffractometer	STOE *IPDS* 2	Bruker *SMART* CCD
Absorption correction	Integration (*X-RED32*; Stoe & Cie, 2002 ▸)	Multi-scan (*SADABS*; Bruker, 2007 ▸)
*T*_min_, *T*_max_	0.899, 0.919	0.861, 0.920
No. of measured, independent and observed [*I* > 2σ(*I*)] reflections	13886, 3287, 2098	14055, 3895, 2611
*R*_int_	0.074	0.040
(sin θ/λ)_max_ (Å^−1^)	0.628	0.668

Refinement		
*R*[*F* ^2^ > 2σ(*F* ^2^)], *wR*(*F* ^2^), *S*	0.049, 0.114, 0.95	0.043, 0.109, 1.04
No. of reflections	3287	3895
No. of parameters	272	272
No. of restraints	61	61
H-atom treatment	H-atom parameters constrained	H-atom parameters constrained
Δρ_max_, Δρ_min_ (e Å^−3^)	0.37, −0.22	0.34, −0.36

Computer programs: *X-AREA* and *X-RED32* (Stoe & Cie, 2002 ▸), *SMART* and *SAINT* (Bruker, 2007 ▸), *SHELXS* (Sheldrick, 2008 ▸), *SHELXL2014* (Sheldrick, 2015 ▸) and *PLATON* (Spek, 2009 ▸).

## supplementary crystallographic information

### Bis(tri-2-pyridylamine)iron(II) bis(perchlorate) (I) Crystal data

**Table:**

[Fe(C_15_H_12_N_4_)_2_](ClO_4_)_2_	*F*(000) = 768
*M**_r_* = 751.32	*D*_x_ = 1.571 Mg m^−^^3^
Monoclinic, *P*2_1_/*n*	Mo *K*α radiation, λ = 0.71073 Å
*a* = 8.3251 (7) Å	Cell parameters from 3322 reflections
*b* = 17.4731 (11) Å	θ = 2.2–26.6°
*c* = 11.0495 (9) Å	µ = 0.71 mm^−^^1^
β = 98.716 (7)°	*T* = 296 K
*V* = 1588.8 (2) Å^3^	Prism, red
*Z* = 2	0.42 × 0.21 × 0.12 mm

### Bis(tri-2-pyridylamine)iron(II) bis(perchlorate) (I) Data collection

**Table:**

STOE IPDS 2 diffractometer	3287 independent reflections
Radiation source: fine focus sealed tube	2098 reflections with *I* > 2σ(*I*)
Plane graphite monochromator	*R*_int_ = 0.074
rotation method scans	θ_max_ = 26.5°, θ_min_ = 2.2°
Absorption correction: integration (X-RED32; Stoe & Cie, 2002)	*h* = −9→10
*T*_min_ = 0.899, *T*_max_ = 0.919	*k* = −21→21
13886 measured reflections	*l* = −13→13

### Bis(tri-2-pyridylamine)iron(II) bis(perchlorate) (I) Refinement

**Table:**

Refinement on *F*^2^	61 restraints
Least-squares matrix: full	Hydrogen site location: inferred from neighbouring sites
*R*[*F*^2^ > 2σ(*F*^2^)] = 0.049	H-atom parameters constrained
*wR*(*F*^2^) = 0.114	*w* = 1/[σ^2^(*F*_o_^2^) + (0.0569*P*)^2^] where *P* = (*F*_o_^2^ + 2*F*_c_^2^)/3
*S* = 0.95	(Δ/σ)_max_ = 0.001
3287 reflections	Δρ_max_ = 0.37 e Å^−^^3^
272 parameters	Δρ_min_ = −0.21 e Å^−^^3^

### Bis(tri-2-pyridylamine)iron(II) bis(perchlorate) (I) Special details

**Table:**

Geometry. All esds (except the esd in the dihedral angle between two l.s. planes) are estimated using the full covariance matrix. The cell esds are taken into account individually in the estimation of esds in distances, angles and torsion angles; correlations between esds in cell parameters are only used when they are defined by crystal symmetry. An approximate (isotropic) treatment of cell esds is used for estimating esds involving l.s. planes.

### Bis(tri-2-pyridylamine)iron(II) bis(perchlorate) (I) Fractional atomic coordinates and isotropic or equivalent isotropic displacement parameters (Å^2^)

**Table:**

	*x*	*y*	*z*	*U*_iso_*/*U*_eq_	Occ. (<1)
Fe1	0.5000	0.5000	0.5000	0.03891 (18)	
N1	0.3758 (3)	0.44032 (16)	0.7164 (2)	0.0448 (6)	
N11	0.3667 (3)	0.55621 (16)	0.6050 (2)	0.0423 (6)	
C12	0.3230 (4)	0.51828 (18)	0.7007 (3)	0.0418 (8)	
C13	0.2334 (4)	0.5506 (2)	0.7821 (3)	0.0549 (9)	
H13	0.2080	0.5228	0.8485	0.066*	
C14	0.1822 (5)	0.6252 (2)	0.7631 (4)	0.0624 (10)	
H14	0.1197	0.6485	0.8157	0.075*	
C15	0.2251 (4)	0.6648 (2)	0.6648 (3)	0.0582 (9)	
H15	0.1917	0.7152	0.6503	0.070*	
C16	0.3174 (4)	0.6291 (2)	0.5889 (3)	0.0493 (8)	
H16	0.3470	0.6565	0.5236	0.059*	
N21	0.3542 (3)	0.41161 (15)	0.5048 (2)	0.0420 (6)	
C22	0.3112 (4)	0.39352 (19)	0.6139 (3)	0.0415 (7)	
C23	0.2113 (4)	0.3321 (2)	0.6298 (3)	0.0504 (8)	
H23	0.1831	0.3214	0.7063	0.061*	
C24	0.1550 (4)	0.2877 (2)	0.5306 (4)	0.0580 (9)	
H24	0.0878	0.2461	0.5387	0.070*	
C25	0.1988 (4)	0.3052 (2)	0.4180 (3)	0.0553 (9)	
H25	0.1625	0.2752	0.3498	0.066*	
C26	0.2966 (4)	0.3674 (2)	0.4084 (3)	0.0473 (8)	
H26	0.3241	0.3794	0.3322	0.057*	
N31	0.6312 (3)	0.45990 (15)	0.6513 (2)	0.0428 (6)	
C32	0.5506 (4)	0.43509 (19)	0.7411 (3)	0.0439 (7)	
C33	0.6251 (5)	0.4056 (2)	0.8493 (3)	0.0568 (9)	
H33	0.5643	0.3899	0.9088	0.068*	
C34	0.7919 (5)	0.3995 (2)	0.8690 (3)	0.0645 (10)	
H34	0.8458	0.3788	0.9416	0.077*	
C35	0.8774 (5)	0.4245 (2)	0.7794 (3)	0.0594 (9)	
H35	0.9902	0.4214	0.7911	0.071*	
C36	0.7957 (4)	0.4539 (2)	0.6731 (3)	0.0504 (8)	
H36	0.8551	0.4705	0.6133	0.061*	
Cl1	0.2289 (10)	0.3486 (6)	0.0675 (8)	0.0622 (9)	0.415 (3)
O11	0.1418 (13)	0.4123 (5)	0.1027 (19)	0.097 (3)	0.415 (3)
O12	0.223 (3)	0.3449 (12)	−0.0609 (8)	0.111 (2)	0.415 (3)
O13	0.3942 (10)	0.3496 (8)	0.1242 (11)	0.101 (4)	0.415 (3)
O14	0.1517 (15)	0.2816 (6)	0.1090 (9)	0.090 (3)	0.415 (3)
Cl2	0.2526 (16)	0.3447 (7)	0.0642 (13)	0.0622 (9)	0.267 (3)
O21	0.120 (2)	0.3833 (9)	0.103 (3)	0.097 (3)	0.267 (3)
O22	0.232 (4)	0.3364 (16)	−0.0645 (12)	0.111 (2)	0.267 (3)
O23	0.4019 (18)	0.3833 (13)	0.104 (2)	0.101 (4)	0.267 (3)
O24	0.261 (2)	0.2702 (7)	0.1209 (13)	0.090 (3)	0.267 (3)
Cl3	0.2227 (13)	0.3347 (8)	0.0565 (11)	0.0622 (9)	0.256 (3)
O31	0.184 (2)	0.4097 (8)	0.090 (3)	0.097 (3)	0.256 (3)
O32	0.261 (3)	0.3320 (18)	−0.0639 (11)	0.111 (2)	0.256 (3)
O33	0.3546 (16)	0.3034 (13)	0.1385 (14)	0.101 (4)	0.256 (3)
O34	0.0812 (17)	0.2883 (10)	0.0635 (16)	0.090 (3)	0.256 (3)
Cl4	0.215 (3)	0.3423 (16)	0.032 (2)	0.0622 (9)	0.061 (3)
O41	0.168 (6)	0.4201 (17)	0.025 (5)	0.097 (3)	0.061 (3)
O42	0.377 (3)	0.332 (3)	0.012 (4)	0.111 (2)	0.061 (3)
O43	0.195 (5)	0.309 (3)	0.147 (3)	0.101 (4)	0.061 (3)
O44	0.107 (5)	0.303 (2)	−0.062 (3)	0.090 (3)	0.061 (3)

### Bis(tri-2-pyridylamine)iron(II) bis(perchlorate) (I) Atomic displacement parameters (Å^2^)

**Table:**

	*U*^11^	*U*^22^	*U*^33^	*U*^12^	*U*^13^	*U*^23^
Fe1	0.0467 (3)	0.0396 (4)	0.0320 (3)	−0.0034 (3)	0.0112 (2)	0.0034 (3)
N1	0.0521 (16)	0.0463 (17)	0.0374 (14)	−0.0063 (13)	0.0116 (12)	0.0021 (12)
N11	0.0504 (15)	0.0400 (16)	0.0376 (14)	−0.0032 (12)	0.0100 (12)	0.0000 (12)
C12	0.0468 (16)	0.044 (2)	0.0352 (15)	−0.0050 (14)	0.0095 (13)	−0.0013 (13)
C13	0.059 (2)	0.064 (3)	0.046 (2)	−0.0069 (19)	0.0209 (16)	−0.0067 (17)
C14	0.061 (2)	0.068 (3)	0.063 (2)	0.004 (2)	0.0243 (19)	−0.014 (2)
C15	0.061 (2)	0.049 (2)	0.065 (2)	0.0080 (18)	0.0099 (18)	−0.0076 (19)
C16	0.055 (2)	0.043 (2)	0.050 (2)	0.0002 (16)	0.0068 (16)	0.0017 (16)
N21	0.0481 (15)	0.0419 (16)	0.0370 (14)	−0.0034 (12)	0.0100 (11)	0.0035 (12)
C22	0.0472 (17)	0.0394 (19)	0.0386 (16)	0.0005 (15)	0.0089 (13)	0.0043 (14)
C23	0.058 (2)	0.045 (2)	0.0506 (19)	−0.0057 (17)	0.0176 (16)	0.0082 (16)
C24	0.060 (2)	0.048 (2)	0.067 (2)	−0.0125 (18)	0.0106 (18)	0.0049 (19)
C25	0.060 (2)	0.050 (2)	0.055 (2)	−0.0109 (17)	0.0029 (16)	−0.0058 (17)
C26	0.056 (2)	0.046 (2)	0.0396 (17)	−0.0057 (16)	0.0065 (14)	−0.0023 (15)
N31	0.0507 (15)	0.0410 (16)	0.0371 (14)	−0.0028 (13)	0.0081 (12)	0.0024 (12)
C32	0.0555 (19)	0.0420 (19)	0.0341 (16)	−0.0018 (15)	0.0061 (14)	0.0024 (14)
C33	0.071 (2)	0.060 (2)	0.0397 (18)	−0.0058 (19)	0.0086 (16)	0.0078 (17)
C34	0.074 (3)	0.068 (3)	0.047 (2)	0.003 (2)	−0.0062 (18)	0.0132 (19)
C35	0.058 (2)	0.059 (2)	0.058 (2)	0.0013 (18)	−0.0022 (17)	0.0021 (18)
C36	0.0514 (19)	0.053 (2)	0.0473 (19)	−0.0037 (17)	0.0094 (15)	0.0013 (17)
Cl1	0.0683 (17)	0.081 (2)	0.0385 (11)	0.0170 (11)	0.0109 (12)	−0.0146 (12)
O11	0.088 (6)	0.072 (5)	0.143 (5)	0.010 (4)	0.058 (5)	−0.036 (6)
O12	0.163 (6)	0.130 (5)	0.0448 (19)	0.013 (5)	0.034 (2)	−0.011 (2)
O13	0.052 (3)	0.163 (15)	0.085 (6)	−0.006 (5)	0.006 (3)	0.020 (9)
O14	0.099 (9)	0.095 (5)	0.075 (5)	−0.001 (6)	0.014 (6)	0.003 (4)
Cl2	0.0683 (17)	0.081 (2)	0.0385 (11)	0.0170 (11)	0.0109 (12)	−0.0146 (12)
O21	0.088 (6)	0.072 (5)	0.143 (5)	0.010 (4)	0.058 (5)	−0.036 (6)
O22	0.163 (6)	0.130 (5)	0.0448 (19)	0.013 (5)	0.034 (2)	−0.011 (2)
O23	0.052 (3)	0.163 (15)	0.085 (6)	−0.006 (5)	0.006 (3)	0.020 (9)
O24	0.099 (9)	0.095 (5)	0.075 (5)	−0.001 (6)	0.014 (6)	0.003 (4)
Cl3	0.0683 (17)	0.081 (2)	0.0385 (11)	0.0170 (11)	0.0109 (12)	−0.0146 (12)
O31	0.088 (6)	0.072 (5)	0.143 (5)	0.010 (4)	0.058 (5)	−0.036 (6)
O32	0.163 (6)	0.130 (5)	0.0448 (19)	0.013 (5)	0.034 (2)	−0.011 (2)
O33	0.052 (3)	0.163 (15)	0.085 (6)	−0.006 (5)	0.006 (3)	0.020 (9)
O34	0.099 (9)	0.095 (5)	0.075 (5)	−0.001 (6)	0.014 (6)	0.003 (4)
Cl4	0.0683 (17)	0.081 (2)	0.0385 (11)	0.0170 (11)	0.0109 (12)	−0.0146 (12)
O41	0.088 (6)	0.072 (5)	0.143 (5)	0.010 (4)	0.058 (5)	−0.036 (6)
O42	0.163 (6)	0.130 (5)	0.0448 (19)	0.013 (5)	0.034 (2)	−0.011 (2)
O43	0.052 (3)	0.163 (15)	0.085 (6)	−0.006 (5)	0.006 (3)	0.020 (9)
O44	0.099 (9)	0.095 (5)	0.075 (5)	−0.001 (6)	0.014 (6)	0.003 (4)

### Bis(tri-2-pyridylamine)iron(II) bis(perchlorate) (I) Geometric parameters (Å, º)

**Table:**

Fe1—N21^i^	1.970 (3)	C26—H26	0.9300
Fe1—N21	1.970 (3)	N31—C32	1.350 (4)
Fe1—N31^i^	1.982 (3)	N31—C36	1.359 (4)
Fe1—N31	1.982 (3)	C32—C33	1.361 (5)
Fe1—N11	1.983 (2)	C33—C34	1.377 (5)
Fe1—N11^i^	1.983 (2)	C33—H33	0.9300
N1—C22	1.433 (4)	C34—C35	1.376 (5)
N1—C12	1.434 (4)	C34—H34	0.9300
N1—C32	1.442 (4)	C35—C36	1.365 (5)
N11—C16	1.342 (4)	C35—H35	0.9300
N11—C12	1.344 (4)	C36—H36	0.9300
C12—C13	1.374 (4)	Cl1—O12	1.413 (4)
C13—C14	1.378 (5)	Cl1—O11	1.416 (5)
C13—H13	0.9300	Cl1—O13	1.423 (6)
C14—C15	1.380 (5)	Cl1—O14	1.443 (6)
C14—H14	0.9300	Cl2—O22	1.413 (5)
C15—C16	1.371 (5)	Cl2—O21	1.414 (5)
C15—H15	0.9300	Cl2—O23	1.424 (7)
C16—H16	0.9300	Cl2—O24	1.441 (7)
N21—C26	1.344 (4)	Cl3—O32	1.414 (5)
N21—C22	1.346 (4)	Cl3—O31	1.414 (5)
C22—C23	1.385 (4)	Cl3—O33	1.423 (7)
C23—C24	1.367 (5)	Cl3—O34	1.442 (7)
C23—H23	0.9300	Cl4—O42	1.413 (6)
C24—C25	1.383 (5)	Cl4—O41	1.415 (6)
C24—H24	0.9300	Cl4—O43	1.423 (7)
C25—C26	1.371 (5)	Cl4—O44	1.442 (7)
C25—H25	0.9300		
			
N21^i^—Fe1—N21	180.0	C26—C25—C24	119.0 (3)
N21^i^—Fe1—N31^i^	87.88 (11)	C26—C25—H25	120.5
N21—Fe1—N31^i^	92.12 (11)	C24—C25—H25	120.5
N21^i^—Fe1—N31	92.11 (11)	N21—C26—C25	122.6 (3)
N21—Fe1—N31	87.89 (11)	N21—C26—H26	118.7
N31^i^—Fe1—N31	180.0	C25—C26—H26	118.7
N21^i^—Fe1—N11	91.67 (10)	C32—N31—C36	116.4 (3)
N21—Fe1—N11	88.33 (10)	C32—N31—Fe1	117.5 (2)
N31^i^—Fe1—N11	91.89 (11)	C36—N31—Fe1	126.1 (2)
N31—Fe1—N11	88.11 (11)	N31—C32—C33	123.7 (3)
N21^i^—Fe1—N11^i^	88.33 (10)	N31—C32—N1	116.1 (3)
N21—Fe1—N11^i^	91.66 (10)	C33—C32—N1	120.2 (3)
N31^i^—Fe1—N11^i^	88.11 (10)	C32—C33—C34	118.9 (3)
N31—Fe1—N11^i^	91.89 (11)	C32—C33—H33	120.6
N11—Fe1—N11^i^	180.0	C34—C33—H33	120.6
C22—N1—C12	112.0 (3)	C35—C34—C33	118.7 (4)
C22—N1—C32	111.1 (2)	C35—C34—H34	120.7
C12—N1—C32	111.4 (3)	C33—C34—H34	120.7
C16—N11—C12	117.3 (3)	C36—C35—C34	119.7 (4)
C16—N11—Fe1	125.4 (2)	C36—C35—H35	120.2
C12—N11—Fe1	117.3 (2)	C34—C35—H35	120.2
N11—C12—C13	123.4 (3)	N31—C36—C35	122.6 (3)
N11—C12—N1	116.7 (2)	N31—C36—H36	118.7
C13—C12—N1	119.9 (3)	C35—C36—H36	118.7
C12—C13—C14	118.4 (3)	O12—Cl1—O11	111.7 (5)
C12—C13—H13	120.8	O12—Cl1—O13	109.2 (5)
C14—C13—H13	120.8	O11—Cl1—O13	111.7 (5)
C13—C14—C15	118.9 (3)	O12—Cl1—O14	109.4 (5)
C13—C14—H14	120.5	O11—Cl1—O14	106.2 (5)
C15—C14—H14	120.5	O13—Cl1—O14	108.5 (6)
C16—C15—C14	119.3 (4)	O22—Cl2—O21	111.9 (6)
C16—C15—H15	120.4	O22—Cl2—O23	109.1 (6)
C14—C15—H15	120.4	O21—Cl2—O23	111.3 (6)
N11—C16—C15	122.7 (3)	O22—Cl2—O24	109.6 (6)
N11—C16—H16	118.7	O21—Cl2—O24	106.6 (6)
C15—C16—H16	118.7	O23—Cl2—O24	108.2 (7)
C26—N21—C22	117.6 (3)	O32—Cl3—O31	111.8 (6)
C26—N21—Fe1	125.2 (2)	O32—Cl3—O33	108.9 (6)
C22—N21—Fe1	117.2 (2)	O31—Cl3—O33	111.8 (7)
N21—C22—C23	122.8 (3)	O32—Cl3—O34	109.5 (6)
N21—C22—N1	117.0 (3)	O31—Cl3—O34	106.7 (6)
C23—C22—N1	120.2 (3)	O33—Cl3—O34	107.9 (7)
C24—C23—C22	118.5 (3)	O42—Cl4—O41	111.8 (7)
C24—C23—H23	120.7	O42—Cl4—O43	109.2 (7)
C22—C23—H23	120.7	O41—Cl4—O43	111.8 (8)
C23—C24—C25	119.4 (3)	O42—Cl4—O44	109.4 (7)
C23—C24—H24	120.3	O41—Cl4—O44	106.4 (7)
C25—C24—H24	120.3	O43—Cl4—O44	108.1 (8)
			
C16—N11—C12—C13	−0.9 (5)	N21—C22—C23—C24	0.5 (5)
Fe1—N11—C12—C13	178.8 (3)	N1—C22—C23—C24	−178.5 (3)
C16—N11—C12—N1	179.7 (3)	C22—C23—C24—C25	−0.1 (5)
Fe1—N11—C12—N1	−0.7 (4)	C23—C24—C25—C26	−0.7 (6)
C22—N1—C12—N11	−61.6 (3)	C22—N21—C26—C25	−0.7 (5)
C32—N1—C12—N11	63.6 (3)	Fe1—N21—C26—C25	178.2 (3)
C22—N1—C12—C13	118.9 (3)	C24—C25—C26—N21	1.1 (5)
C32—N1—C12—C13	−115.9 (3)	C36—N31—C32—C33	0.3 (5)
N11—C12—C13—C14	1.7 (6)	Fe1—N31—C32—C33	179.5 (3)
N1—C12—C13—C14	−178.9 (3)	C36—N31—C32—N1	−178.9 (3)
C12—C13—C14—C15	−1.2 (6)	Fe1—N31—C32—N1	0.2 (4)
C13—C14—C15—C16	−0.1 (6)	C22—N1—C32—N31	62.5 (4)
C12—N11—C16—C15	−0.5 (5)	C12—N1—C32—N31	−63.2 (3)
Fe1—N11—C16—C15	180.0 (3)	C22—N1—C32—C33	−116.7 (3)
C14—C15—C16—N11	0.9 (6)	C12—N1—C32—C33	117.5 (3)
C26—N21—C22—C23	−0.1 (5)	N31—C32—C33—C34	−0.9 (6)
Fe1—N21—C22—C23	−179.1 (3)	N1—C32—C33—C34	178.3 (3)
C26—N21—C22—N1	178.9 (3)	C32—C33—C34—C35	1.0 (6)
Fe1—N21—C22—N1	−0.1 (4)	C33—C34—C35—C36	−0.6 (6)
C12—N1—C22—N21	62.3 (3)	C32—N31—C36—C35	0.1 (5)
C32—N1—C22—N21	−63.0 (4)	Fe1—N31—C36—C35	−179.0 (3)
C12—N1—C22—C23	−118.6 (3)	C34—C35—C36—N31	0.1 (6)
C32—N1—C22—C23	116.0 (3)		

Symmetry code: (i) −*x*+1, −*y*+1, −*z*+1.

### Bis(tri-2-pyridylamine)iron(II) bis(perchlorate) (I) Hydrogen-bond geometry (Å, º)

**Table:**

*D*—H···*A*	*D*—H	H···*A*	*D*···*A*	*D*—H···*A*
C14—H14···O21^ii^	0.93	2.38	3.11 (2)	136
C14—H14···O34^ii^	0.93	2.54	3.470 (17)	173
C15—H15···O22^iii^	0.93	2.44	3.24 (3)	143
C15—H15···O32^iii^	0.93	2.31	3.14 (3)	147
C23—H23···O12^iv^	0.93	2.58	3.412 (10)	150
C23—H23···O22^iv^	0.93	2.52	3.357 (14)	150
C23—H23···O32^iv^	0.93	2.53	3.347 (12)	147
C24—H24···O13^v^	0.93	2.60	3.496 (12)	163
C24—H24···O33^v^	0.93	2.53	3.334 (17)	145
C24—H24···O42^v^	0.93	2.21	3.10 (4)	161
C26—H26···O13	0.93	2.51	3.375 (12)	155
C26—H26···O33	0.93	2.56	3.289 (17)	135
C33—H33···O42^iv^	0.93	2.30	3.21 (4)	164

Symmetry codes: (ii) −*x*, −*y*+1, −*z*+1; (iii) −*x*+1/2, *y*+1/2, −*z*+1/2; (iv) *x*, *y*, *z*+1; (v) *x*−1/2, −*y*+1/2, *z*+1/2.

### Bis(tri-2-pyridylamine)nickel(II) bis(perchlorate) (III) Crystal data

**Table:**

[Ni(C_15_H_12_N_4_)_2_](ClO_4_)_2_	*F*(000) = 772
*M**_r_* = 754.18	*D*_x_ = 1.549 Mg m^−^^3^
Monoclinic, *P*2_1_/*n*	Mo *K*α radiation, λ = 0.71073 Å
*a* = 8.360 (4) Å	Cell parameters from 2895 reflections
*b* = 17.570 (8) Å	θ = 2.3–28.4°
*c* = 11.165 (5) Å	µ = 0.83 mm^−^^1^
β = 99.542 (5)°	*T* = 296 K
*V* = 1617.3 (13) Å^3^	Block, purple
*Z* = 2	0.22 × 0.15 × 0.10 mm

### Bis(tri-2-pyridylamine)nickel(II) bis(perchlorate) (III) Data collection

**Table:**

Bruker SMART CCD diffractometer	3895 independent reflections
Radiation source: fine focus sealed tube	2611 reflections with *I* > 2σ(*I*)
Graphite monochromator	*R*_int_ = 0.040
φ and ω scans	θ_max_ = 28.4°, θ_min_ = 2.3°
Absorption correction: multi-scan (SADABS; Bruker, 2007)	*h* = −10→10
*T*_min_ = 0.861, *T*_max_ = 0.920	*k* = −22→22
14055 measured reflections	*l* = −14→14

### Bis(tri-2-pyridylamine)nickel(II) bis(perchlorate) (III) Refinement

**Table:**

Refinement on *F*^2^	61 restraints
Least-squares matrix: full	Hydrogen site location: inferred from neighbouring sites
*R*[*F*^2^ > 2σ(*F*^2^)] = 0.043	H-atom parameters constrained
*wR*(*F*^2^) = 0.109	*w* = 1/[σ^2^(*F*_o_^2^) + (0.0471*P*)^2^ + 0.3274*P*] where *P* = (*F*_o_^2^ + 2*F*_c_^2^)/3
*S* = 1.04	(Δ/σ)_max_ < 0.001
3895 reflections	Δρ_max_ = 0.34 e Å^−^^3^
272 parameters	Δρ_min_ = −0.36 e Å^−^^3^

### Bis(tri-2-pyridylamine)nickel(II) bis(perchlorate) (III) Special details

**Table:**

Geometry. All esds (except the esd in the dihedral angle between two l.s. planes) are estimated using the full covariance matrix. The cell esds are taken into account individually in the estimation of esds in distances, angles and torsion angles; correlations between esds in cell parameters are only used when they are defined by crystal symmetry. An approximate (isotropic) treatment of cell esds is used for estimating esds involving l.s. planes.

### Bis(tri-2-pyridylamine)nickel(II) bis(perchlorate) (III) Fractional atomic coordinates and isotropic or equivalent isotropic displacement parameters (Å^2^)

**Table:**

	*x*	*y*	*z*	*U*_iso_*/*U*_eq_	Occ. (<1)
Ni1	0.5000	0.5000	0.5000	0.03796 (15)	
N1	0.3741 (3)	0.44195 (11)	0.71780 (17)	0.0413 (5)	
N11	0.3603 (3)	0.55818 (11)	0.60850 (17)	0.0425 (5)	
C12	0.3193 (3)	0.51938 (14)	0.7020 (2)	0.0402 (6)	
C13	0.2283 (3)	0.55033 (16)	0.7809 (2)	0.0523 (7)	
H13	0.2027	0.5220	0.8455	0.063*	
C14	0.1757 (4)	0.62410 (17)	0.7626 (3)	0.0618 (8)	
H14	0.1120	0.6463	0.8139	0.074*	
C15	0.2179 (3)	0.66449 (16)	0.6685 (3)	0.0571 (7)	
H15	0.1843	0.7147	0.6555	0.068*	
C16	0.3101 (3)	0.63049 (15)	0.5931 (2)	0.0491 (6)	
H16	0.3388	0.6585	0.5292	0.059*	
N21	0.3472 (3)	0.40791 (11)	0.50938 (17)	0.0421 (5)	
C22	0.3077 (3)	0.39311 (13)	0.6185 (2)	0.0398 (6)	
C23	0.2089 (3)	0.33333 (14)	0.6379 (2)	0.0487 (6)	
H23	0.1817	0.3248	0.7143	0.058*	
C24	0.1512 (4)	0.28645 (16)	0.5418 (3)	0.0586 (7)	
H24	0.0852	0.2453	0.5526	0.070*	
C25	0.1917 (3)	0.30088 (15)	0.4301 (3)	0.0544 (7)	
H25	0.1539	0.2697	0.3642	0.065*	
C26	0.2887 (3)	0.36191 (15)	0.4173 (2)	0.0494 (6)	
H26	0.3151	0.3718	0.3411	0.059*	
N31	0.6341 (3)	0.45832 (11)	0.66281 (17)	0.0421 (5)	
C32	0.5477 (3)	0.43591 (13)	0.7471 (2)	0.0410 (6)	
C33	0.6174 (4)	0.40688 (16)	0.8578 (2)	0.0547 (7)	
H33	0.5540	0.3925	0.9150	0.066*	
C34	0.7836 (4)	0.39971 (17)	0.8818 (3)	0.0642 (8)	
H34	0.8342	0.3799	0.9557	0.077*	
C35	0.8739 (4)	0.42181 (16)	0.7965 (3)	0.0573 (7)	
H35	0.9863	0.4170	0.8111	0.069*	
C36	0.7953 (3)	0.45125 (16)	0.6888 (2)	0.0531 (7)	
H36	0.8570	0.4670	0.6313	0.064*	
Cl1	0.2327 (9)	0.3463 (5)	0.0711 (7)	0.0603 (7)	0.424 (3)
O11	0.1382 (11)	0.4106 (4)	0.0898 (16)	0.104 (2)	0.424 (3)
O12	0.238 (2)	0.3337 (11)	−0.0522 (7)	0.1217 (17)	0.424 (3)
O13	0.3919 (9)	0.3541 (7)	0.1374 (10)	0.104 (3)	0.424 (3)
O14	0.1568 (12)	0.2813 (4)	0.1163 (8)	0.095 (2)	0.424 (3)
Cl2	0.2562 (14)	0.3409 (6)	0.0680 (11)	0.0603 (7)	0.280 (3)
O21	0.1225 (17)	0.3830 (7)	0.095 (2)	0.104 (2)	0.280 (3)
O22	0.246 (4)	0.3265 (14)	−0.0566 (11)	0.1217 (17)	0.280 (3)
O23	0.4031 (15)	0.3795 (11)	0.113 (2)	0.104 (3)	0.280 (3)
O24	0.2573 (17)	0.2693 (6)	0.1296 (11)	0.095 (2)	0.280 (3)
Cl3	0.2238 (12)	0.3356 (7)	0.0680 (11)	0.0603 (7)	0.244 (3)
O31	0.190 (2)	0.4077 (7)	0.114 (3)	0.104 (2)	0.244 (3)
O32	0.260 (3)	0.340 (2)	−0.0501 (11)	0.1217 (17)	0.244 (3)
O33	0.3537 (13)	0.3010 (10)	0.1470 (11)	0.104 (3)	0.244 (3)
O34	0.0822 (14)	0.2890 (8)	0.0645 (13)	0.095 (2)	0.244 (3)
Cl4	0.196 (2)	0.3332 (12)	0.0342 (16)	0.0603 (7)	0.052 (2)
O41	0.177 (5)	0.4126 (12)	0.016 (4)	0.104 (2)	0.052 (2)
O42	0.356 (3)	0.309 (2)	0.034 (3)	0.1217 (17)	0.052 (2)
O43	0.143 (5)	0.312 (3)	0.143 (2)	0.104 (3)	0.052 (2)
O44	0.094 (4)	0.296 (2)	−0.065 (3)	0.095 (2)	0.052 (2)

### Bis(tri-2-pyridylamine)nickel(II) bis(perchlorate) (III) Atomic displacement parameters (Å^2^)

**Table:**

	*U*^11^	*U*^22^	*U*^33^	*U*^12^	*U*^13^	*U*^23^
Ni1	0.0482 (3)	0.0388 (2)	0.0288 (2)	−0.0034 (2)	0.01177 (18)	0.00442 (18)
N1	0.0527 (13)	0.0397 (11)	0.0330 (11)	−0.0033 (10)	0.0114 (9)	0.0027 (9)
N11	0.0527 (13)	0.0418 (11)	0.0348 (11)	0.0006 (9)	0.0122 (9)	0.0028 (9)
C12	0.0432 (14)	0.0467 (14)	0.0318 (12)	−0.0051 (11)	0.0095 (11)	−0.0012 (10)
C13	0.0587 (18)	0.0594 (17)	0.0427 (15)	−0.0064 (14)	0.0201 (13)	−0.0062 (13)
C14	0.0608 (19)	0.0673 (19)	0.0622 (19)	0.0041 (15)	0.0244 (15)	−0.0156 (16)
C15	0.0600 (18)	0.0490 (16)	0.0617 (18)	0.0085 (14)	0.0088 (15)	−0.0076 (14)
C16	0.0579 (17)	0.0438 (14)	0.0459 (15)	0.0018 (12)	0.0096 (13)	0.0041 (12)
N21	0.0543 (13)	0.0413 (11)	0.0309 (10)	−0.0060 (10)	0.0080 (9)	0.0037 (9)
C22	0.0456 (14)	0.0384 (13)	0.0359 (13)	−0.0011 (11)	0.0085 (11)	0.0067 (10)
C23	0.0529 (16)	0.0467 (14)	0.0485 (15)	−0.0042 (12)	0.0143 (13)	0.0101 (12)
C24	0.0600 (18)	0.0470 (15)	0.0686 (19)	−0.0157 (13)	0.0097 (15)	0.0025 (14)
C25	0.0588 (18)	0.0474 (15)	0.0547 (17)	−0.0076 (13)	0.0026 (14)	−0.0058 (13)
C26	0.0599 (18)	0.0515 (15)	0.0371 (14)	−0.0048 (13)	0.0085 (12)	−0.0006 (12)
N31	0.0477 (13)	0.0450 (12)	0.0336 (11)	−0.0035 (10)	0.0070 (9)	0.0046 (9)
C32	0.0534 (16)	0.0385 (13)	0.0315 (12)	−0.0019 (11)	0.0085 (11)	0.0034 (10)
C33	0.069 (2)	0.0583 (17)	0.0361 (14)	−0.0048 (15)	0.0079 (13)	0.0108 (12)
C34	0.072 (2)	0.070 (2)	0.0447 (16)	0.0036 (17)	−0.0072 (15)	0.0123 (14)
C35	0.0529 (18)	0.0607 (18)	0.0548 (18)	0.0014 (14)	−0.0015 (14)	0.0024 (14)
C36	0.0531 (18)	0.0570 (17)	0.0493 (16)	−0.0028 (14)	0.0087 (13)	0.0060 (13)
Cl1	0.0585 (17)	0.0840 (14)	0.0389 (6)	0.0191 (10)	0.0099 (9)	−0.0132 (8)
O11	0.081 (5)	0.076 (4)	0.156 (6)	0.012 (3)	0.025 (5)	−0.052 (5)
O12	0.184 (4)	0.142 (4)	0.0458 (16)	−0.004 (3)	0.039 (2)	−0.019 (2)
O13	0.053 (3)	0.177 (10)	0.080 (5)	−0.015 (4)	0.003 (3)	0.010 (6)
O14	0.104 (8)	0.099 (4)	0.086 (5)	0.015 (5)	0.024 (6)	0.015 (3)
Cl2	0.0585 (17)	0.0840 (14)	0.0389 (6)	0.0191 (10)	0.0099 (9)	−0.0132 (8)
O21	0.081 (5)	0.076 (4)	0.156 (6)	0.012 (3)	0.025 (5)	−0.052 (5)
O22	0.184 (4)	0.142 (4)	0.0458 (16)	−0.004 (3)	0.039 (2)	−0.019 (2)
O23	0.053 (3)	0.177 (10)	0.080 (5)	−0.015 (4)	0.003 (3)	0.010 (6)
O24	0.104 (8)	0.099 (4)	0.086 (5)	0.015 (5)	0.024 (6)	0.015 (3)
Cl3	0.0585 (17)	0.0840 (14)	0.0389 (6)	0.0191 (10)	0.0099 (9)	−0.0132 (8)
O31	0.081 (5)	0.076 (4)	0.156 (6)	0.012 (3)	0.025 (5)	−0.052 (5)
O32	0.184 (4)	0.142 (4)	0.0458 (16)	−0.004 (3)	0.039 (2)	−0.019 (2)
O33	0.053 (3)	0.177 (10)	0.080 (5)	−0.015 (4)	0.003 (3)	0.010 (6)
O34	0.104 (8)	0.099 (4)	0.086 (5)	0.015 (5)	0.024 (6)	0.015 (3)
Cl4	0.0585 (17)	0.0840 (14)	0.0389 (6)	0.0191 (10)	0.0099 (9)	−0.0132 (8)
O41	0.081 (5)	0.076 (4)	0.156 (6)	0.012 (3)	0.025 (5)	−0.052 (5)
O42	0.184 (4)	0.142 (4)	0.0458 (16)	−0.004 (3)	0.039 (2)	−0.019 (2)
O43	0.053 (3)	0.177 (10)	0.080 (5)	−0.015 (4)	0.003 (3)	0.010 (6)
O44	0.104 (8)	0.099 (4)	0.086 (5)	0.015 (5)	0.024 (6)	0.015 (3)

### Bis(tri-2-pyridylamine)nickel(II) bis(perchlorate) (III) Geometric parameters (Å, º)

**Table:**

Ni1—N21	2.075 (2)	C26—H26	0.9300
Ni1—N21^i^	2.075 (2)	N31—C36	1.336 (3)
Ni1—N11	2.084 (2)	N31—C32	1.337 (3)
Ni1—N11^i^	2.085 (2)	C32—C33	1.374 (3)
Ni1—N31	2.103 (2)	C33—C34	1.377 (4)
Ni1—N31^i^	2.103 (2)	C33—H33	0.9300
N1—C12	1.437 (3)	C34—C35	1.365 (4)
N1—C32	1.438 (3)	C34—H34	0.9300
N1—C22	1.439 (3)	C35—C36	1.373 (4)
N11—C12	1.338 (3)	C35—H35	0.9300
N11—C16	1.340 (3)	C36—H36	0.9300
C12—C13	1.369 (3)	Cl1—O12	1.402 (4)
C13—C14	1.373 (4)	Cl1—O11	1.415 (4)
C13—H13	0.9300	Cl1—O13	1.419 (5)
C14—C15	1.362 (4)	Cl1—O14	1.438 (5)
C14—H14	0.9300	Cl2—O22	1.402 (4)
C15—C16	1.369 (4)	Cl2—O21	1.412 (5)
C15—H15	0.9300	Cl2—O23	1.419 (5)
C16—H16	0.9300	Cl2—O24	1.434 (6)
N21—C26	1.335 (3)	Cl3—O32	1.402 (4)
N21—C22	1.339 (3)	Cl3—O31	1.413 (5)
C22—C23	1.376 (3)	Cl3—O33	1.418 (6)
C23—C24	1.375 (4)	Cl3—O34	1.434 (6)
C23—H23	0.9300	Cl4—O42	1.401 (5)
C24—C25	1.368 (4)	Cl4—O41	1.415 (5)
C24—H24	0.9300	Cl4—O43	1.417 (6)
C25—C26	1.366 (4)	Cl4—O44	1.435 (6)
C25—H25	0.9300		
			
N21—Ni1—N21^i^	180.0	C26—C25—C24	118.8 (3)
N21—Ni1—N11	86.80 (8)	C26—C25—H25	120.6
N21^i^—Ni1—N11	93.20 (8)	C24—C25—H25	120.6
N21—Ni1—N11^i^	93.20 (8)	N21—C26—C25	122.8 (2)
N21^i^—Ni1—N11^i^	86.80 (8)	N21—C26—H26	118.6
N11—Ni1—N11^i^	180.0	C25—C26—H26	118.6
N21—Ni1—N31	85.94 (8)	C36—N31—C32	117.5 (2)
N21^i^—Ni1—N31	94.06 (8)	C36—N31—Ni1	126.46 (17)
N11—Ni1—N31	86.46 (8)	C32—N31—Ni1	116.00 (17)
N11^i^—Ni1—N31	93.54 (8)	N31—C32—C33	123.0 (3)
N21—Ni1—N31^i^	94.06 (8)	N31—C32—N1	117.4 (2)
N21^i^—Ni1—N31^i^	85.94 (8)	C33—C32—N1	119.5 (2)
N11—Ni1—N31^i^	93.54 (8)	C32—C33—C34	118.2 (3)
N11^i^—Ni1—N31^i^	86.46 (8)	C32—C33—H33	120.9
N31—Ni1—N31^i^	180.0	C34—C33—H33	120.9
C12—N1—C32	112.76 (18)	C35—C34—C33	119.7 (3)
C12—N1—C22	113.31 (19)	C35—C34—H34	120.1
C32—N1—C22	112.13 (19)	C33—C34—H34	120.1
C12—N11—C16	117.9 (2)	C34—C35—C36	118.5 (3)
C12—N11—Ni1	116.37 (16)	C34—C35—H35	120.7
C16—N11—Ni1	125.75 (17)	C36—C35—H35	120.7
N11—C12—C13	122.8 (2)	N31—C36—C35	123.0 (3)
N11—C12—N1	117.4 (2)	N31—C36—H36	118.5
C13—C12—N1	119.8 (2)	C35—C36—H36	118.5
C12—C13—C14	118.5 (2)	O12—Cl1—O11	112.3 (4)
C12—C13—H13	120.7	O12—Cl1—O13	110.3 (5)
C14—C13—H13	120.7	O11—Cl1—O13	110.1 (5)
C15—C14—C13	119.3 (3)	O12—Cl1—O14	107.9 (5)
C15—C14—H14	120.4	O11—Cl1—O14	107.1 (4)
C13—C14—H14	120.4	O13—Cl1—O14	108.9 (5)
C14—C15—C16	119.4 (3)	O22—Cl2—O21	112.5 (5)
C14—C15—H15	120.3	O22—Cl2—O23	110.1 (6)
C16—C15—H15	120.3	O21—Cl2—O23	110.1 (6)
N11—C16—C15	122.1 (2)	O22—Cl2—O24	108.2 (6)
N11—C16—H16	118.9	O21—Cl2—O24	107.5 (6)
C15—C16—H16	118.9	O23—Cl2—O24	108.3 (6)
C26—N21—C22	118.0 (2)	O32—Cl3—O31	112.3 (6)
C26—N21—Ni1	125.66 (17)	O32—Cl3—O33	110.3 (6)
C22—N21—Ni1	116.35 (16)	O31—Cl3—O33	109.7 (6)
N21—C22—C23	122.5 (2)	O32—Cl3—O34	108.1 (6)
N21—C22—N1	117.6 (2)	O31—Cl3—O34	107.9 (6)
C23—C22—N1	119.9 (2)	O33—Cl3—O34	108.5 (6)
C24—C23—C22	118.4 (2)	O42—Cl4—O41	112.3 (7)
C24—C23—H23	120.8	O42—Cl4—O43	110.8 (7)
C22—C23—H23	120.8	O41—Cl4—O43	110.0 (7)
C25—C24—C23	119.5 (2)	O42—Cl4—O44	108.2 (7)
C25—C24—H24	120.3	O41—Cl4—O44	107.2 (7)
C23—C24—H24	120.3	O43—Cl4—O44	108.2 (7)
			
C16—N11—C12—C13	0.4 (4)	N21—C22—C23—C24	1.2 (4)
Ni1—N11—C12—C13	179.8 (2)	N1—C22—C23—C24	−178.2 (2)
C16—N11—C12—N1	−179.9 (2)	C22—C23—C24—C25	−0.7 (4)
Ni1—N11—C12—N1	−0.4 (3)	C23—C24—C25—C26	−0.1 (4)
C32—N1—C12—N11	65.2 (3)	C22—N21—C26—C25	−0.2 (4)
C22—N1—C12—N11	−63.5 (3)	Ni1—N21—C26—C25	178.2 (2)
C32—N1—C12—C13	−115.0 (3)	C24—C25—C26—N21	0.6 (4)
C22—N1—C12—C13	116.2 (3)	C36—N31—C32—C33	0.4 (4)
N11—C12—C13—C14	0.6 (4)	Ni1—N31—C32—C33	179.5 (2)
N1—C12—C13—C14	−179.1 (2)	C36—N31—C32—N1	−178.6 (2)
C12—C13—C14—C15	−1.2 (4)	Ni1—N31—C32—N1	0.5 (3)
C13—C14—C15—C16	0.8 (5)	C12—N1—C32—N31	−65.0 (3)
C12—N11—C16—C15	−0.9 (4)	C22—N1—C32—N31	64.3 (3)
Ni1—N11—C16—C15	179.8 (2)	C12—N1—C32—C33	115.9 (2)
C14—C15—C16—N11	0.3 (4)	C22—N1—C32—C33	−114.8 (2)
C26—N21—C22—C23	−0.7 (4)	N31—C32—C33—C34	−0.9 (4)
Ni1—N21—C22—C23	−179.29 (19)	N1—C32—C33—C34	178.1 (2)
C26—N21—C22—N1	178.7 (2)	C32—C33—C34—C35	0.5 (4)
Ni1—N21—C22—N1	0.1 (3)	C33—C34—C35—C36	0.4 (5)
C12—N1—C22—N21	63.9 (3)	C32—N31—C36—C35	0.5 (4)
C32—N1—C22—N21	−65.1 (3)	Ni1—N31—C36—C35	−178.5 (2)
C12—N1—C22—C23	−116.7 (2)	C34—C35—C36—N31	−1.0 (4)
C32—N1—C22—C23	114.3 (2)		

Symmetry code: (i) −*x*+1, −*y*+1, −*z*+1.

### Bis(tri-2-pyridylamine)nickel(II) bis(perchlorate) (III) Hydrogen-bond geometry (Å, º)

**Table:**

*D*—H···*A*	*D*—H	H···*A*	*D*···*A*	*D*—H···*A*
C14—H14···O21^ii^	0.93	2.41	3.174 (17)	139
C14—H14···O34^ii^	0.93	2.55	3.477 (14)	174
C14—H14···O43^ii^	0.93	2.38	3.23 (4)	151
C15—H15···O12^iii^	0.93	2.52	3.289 (18)	140
C15—H15···O22^iii^	0.93	2.37	3.14 (3)	140
C15—H15···O32^iii^	0.93	2.57	3.37 (3)	144
C23—H23···O12^iv^	0.93	2.58	3.428 (8)	152
C23—H23···O22^iv^	0.93	2.53	3.376 (13)	152
C24—H24···O33^v^	0.93	2.49	3.301 (13)	146
C24—H24···O42^v^	0.93	2.12	2.97 (3)	152
C26—H26···O13	0.93	2.48	3.380 (11)	162
C26—H26···O33	0.93	2.57	3.331 (13)	140

Symmetry codes: (ii) −*x*, −*y*+1, −*z*+1; (iii) −*x*+1/2, *y*+1/2, −*z*+1/2; (iv) *x*, *y*, *z*+1; (v) *x*−1/2, −*y*+1/2, *z*+1/2.
